# Case Report of a Traumatic Atlantoaxial Rotatory Subluxation with Bilateral Locked Cervical Facets: Management, Treatment, and Outcome

**DOI:** 10.1155/2016/7308653

**Published:** 2016-03-28

**Authors:** Nael Hawi, Dirk Alfke, Emmanouil Liodakis, Mohamed Omar, Christian Krettek, Christian Walter Müller, Rupert Meller

**Affiliations:** ^1^Trauma Department, Hannover Medical School, Carl-Neuberg-Straße 1, 30625 Hannover, Germany; ^2^Department of Diagnostic and Interventional Radiology, Hannover Medical School, Carl-Neuberg-Straße 1, 30625 Hannover, Germany

## Abstract

The aim was to report a rare case of isolated traumatic atlantoaxial rotatory subluxation without ligamentous injury. Management consisted of analgesia, sedation, and application of a halo skull traction device. After removing halo skull traction, full reduction and recovery were achieved without instability.

## 1. Introduction

Isolated traumatic atlantoaxial rotatory subluxation in adults represents a rare entity. Few cases about subluxation and dislocation have been described in the literature. We define the term subluxation similar to the definition used by Venkatesan et al. [1]: a partial and transient reducible displacement of the adjacent articular surfaces at this level.

We present a rare case of traumatic atlantoaxial rotatory subluxation with first presented clinical (video/photo) and radiological (computed tomography/magnetic resonance) imaging of examination, management, treatment, and outcome.

## 2. Case Report

A fit 34-year-old woman presented after an automobile accident: a head-on collision as driver at a speed of 43.5 mph (70 km/h). There was no loss of consciousness, nausea, or vertigo after the accident.

The patient's neck was stabilized at the accident site (routine procedure with a stiff neck) and she was conveyed to the hospital. She had isolated pain in the cervical neck, with a combination of torticollis, a typical cock robin position of the head, and motion-related pain in the cervical neck with loss of range of motion to the left side (Video 1 in Supplementary Material available online at http://dx.doi.org/10.1155/2016/7308653). She exhibited cervicooccipital tenderness. There was no neurological deficit. She had no previous history concerning cervical spine injuries.

After conventional radiological imaging of the upper spine, computed tomography revealed atlantoaxial rotatory subluxation. The facet of the atlas of one side was dislocated anteriorly over the facet of the axis, whereas the contralateral facet was dislocated/subluxated posteriorly over the facet of the axis. The atlantodental interval was not apparently widened, and there were no associated fractures.

**Figure 1:**
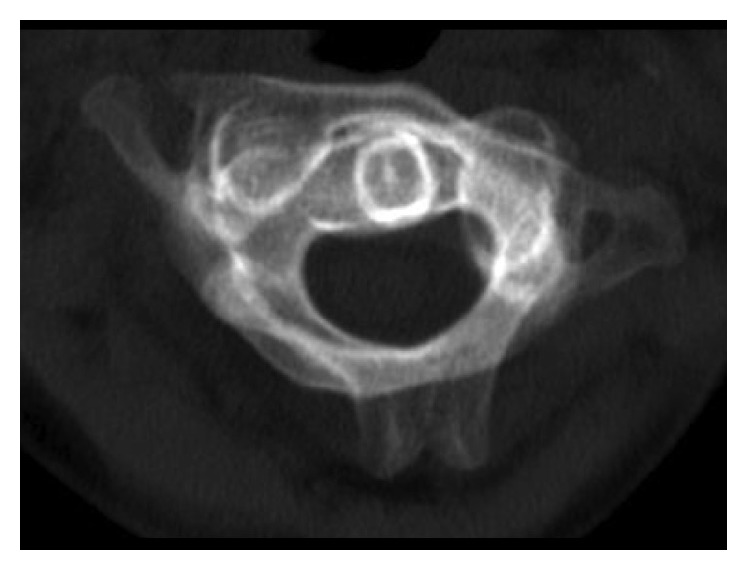
The initial axial CT scan of C1-C2 demonstrates rotatory displacement, CT-MIP (maximum intensity projection).

**Figure 2 fig2:**
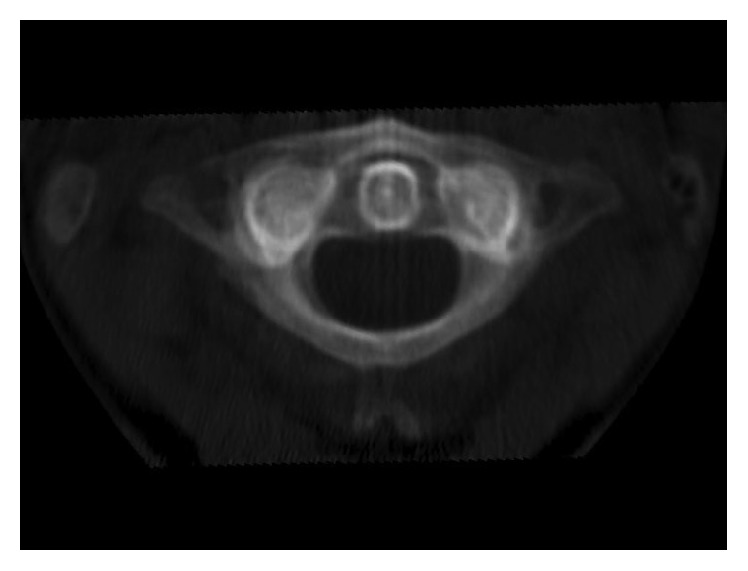
This axial CT scan depicts C1-C2 after reduction, CT-MIP (maximum intensity projection).

**Figure 3 fig3:**
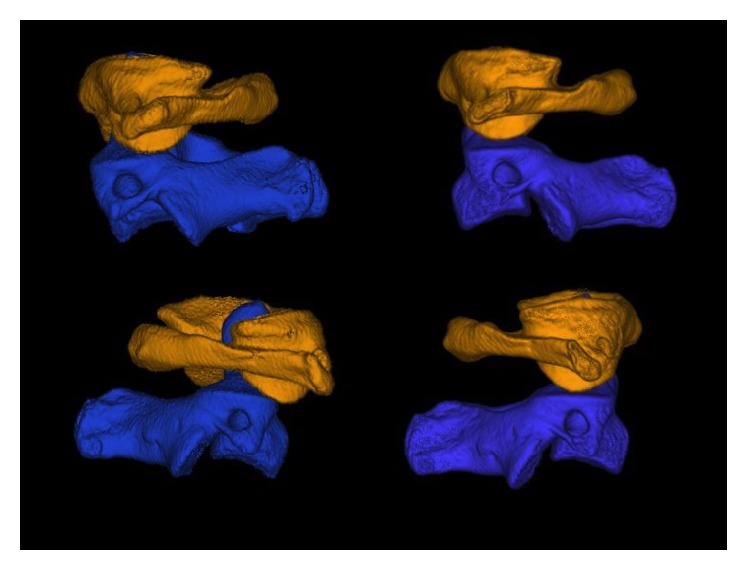
Lateral view from both sides before and after reduction in CT-VRT with segmentation of C1 and C2 (volume rendering technique).

**Figure 4 fig4:**
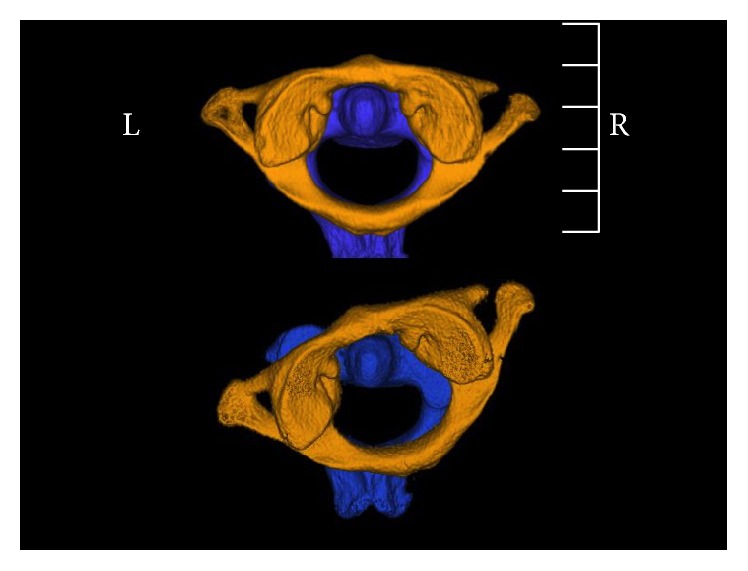
Cranial view after and before reduction in CT-VRT with segmentation of C1 and C2.

**Figure 5:**
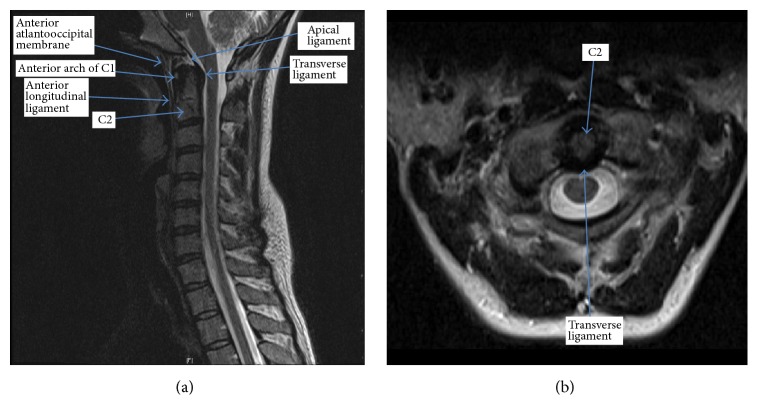
Sagittal (a) and axial (b) MRI images depicting the ligaments of the upper cervical spine.

Management consisted of analgesia (0.3 mg fentanyl), sedation (3 mg midazolam), and application of a halo skull traction device under fluoroscopy (10 lbs, 4.5 kg). After application of the halo skull traction device, computed tomography and magnetic resonance imaging were performed; they revealed full reduction of the subluxation without any ligamentous injuries (Figures 1 [Fig fig2] [Fig fig3] [Fig fig4]–5). After 2 weeks, halo skull traction was removed, and pain-free and nearly free motion of the neck was observed (Video 1). The patient's neck was immobilized with a rigid collar for the next 6 weeks.

At the end of follow-up (6 months), the patient demonstrated free motion of the neck without instability, no pain during motion, and full return to normal activities (Video 1).

## 3. Discussion

Rotation in the cervical spine is mainly based on the atlantoaxial joint, wherein the transverse and alar ligaments provide ligamentous stability. The transverse ligament and the atlantoaxial facet joint capsule prevent anterior dislocation. The alar ligaments typically prevent anterior shift of the atlas and excessive atlantoaxial rotation [2]. Cadaveric studies suggest disruption of the facet capsule followed by the alar ligament [3, 4] as a pathologic mechanism underlying subluxation. In severe forms of rotatory instability, the lateral mass of the atlas locks behind the ipsilateral mass.

Atlantoaxial rotatory subluxation or dislocation usually occurs in pediatric patients with ligamentous laxity, Down's syndrome, inflammatory rheumatoid arthritis, Grisel's syndrome, or congenital anatomical abnormalities [5–11]. In contrast, traumatic atlantoaxial subluxation/dislocation in adults has rarely been described in the literature. Traumatic mechanisms range from motor vehicle accidents and sports-related injuries to falls from great heights [1, 12–18].

As in our present case, typical clinical presentation includes pain in the cervical spine and torticollis. Neurological deficit varies from case to case and depends on the integrity of the dens, the transverse ligaments, and the lesion of the spinal cord [3, 19]. Some cases have reported associated fractures of the lateral mass and odontoid process [20, 21].

The Fielding classification system is the commonly used system for classifying rotatory atlantoaxial instabilities [8].

After clinical examination, radiological assessment should include plain radiographs and computed tomography of the occipitocervical segment. Atlantoaxial asymmetry after trauma can have different causes [22, 23]. Magnetic resonance imaging can be employed to evaluate the transverse ligament, alar ligaments, and spinal cord [4, 24].

Therapeutic management of this type of injury is still controversial because the injury is so rare. In general, prompt reduction and stabilization are mandatory. Whether the method of stabilization is external or internal depends mainly on stable and anatomic atlantoaxial reduction. In cases of fractures, neurological deficit, ligamentous disruption, or nonanatomic reduction through traction internal fixation should be considered [8, 13, 20].

We present a rare case of Fielding type I atlantoaxial rotatory instability with complete recovery of range of motion, recovery of stability, and full decline of pain. In our case, prompt halo skull traction for 14 days followed by a rigid collar for the next 6 weeks was sufficient to achieve good and pain-free rotational stability. Magnetic resonance imaging was performed after traction and did not reveal any ligamentous damage. Follow-up flexion-extension radiographs did not reveal any signs of ligamentous instability.

## Supplementary Material

Video 1: Preoperative and postoperative range of motion of the cervical spine.
